# Sirt6 depletion causes spindle defects and chromosome misalignment during meiosis of mouse oocyte

**DOI:** 10.1038/srep15366

**Published:** 2015-10-20

**Authors:** Longsen Han, Juan Ge, Liang Zhang, Rujun Ma, Xiaojing Hou, Bin Li, Kelle Moley, Qiang Wang

**Affiliations:** 1 State Key Laboratory of Reproductive Medicine, Nanjing Medical University, Nanjing 210029, China; 2 College of Animal Science and Technology, Nanjing Agricultural University, Nanjing, 210095, China; 3 Center of Reproductive Medicine, Nanjing Jinling Hospital, Nanjing University School of Medicine, Nanjing, 210002, China; 4 School of Police Dog Technique of The Ministry of Public Security, Shenyang, 110034, China.; 5 Department of Obstetrics and Gynecology, Washington University School of Medicine, St Louis, MO 63110, USA

## Abstract

Sirt6, a member of the sirtuin family of NAD-dependent protein deacetylases, has been implicated in multiple biological processes. However, the roles of Sirt6 in meiosis have not been addressed. In the present study, by employing knockdown analysis in mouse oocytes, we evaluated the effects of Sirt6 on meiotic apparatus. We found that specific depletion of Sirt6 results in disruption of spindle morphology and chromosome alignment in oocytes. Consistent with this observation, incidence of aneuploidy is also markedly increased in Sirt6-depleted oocytes. Furthermore, confocal scanning showed that kinetochore-microtubule interaction, an important mechanism controlling chromosome segregation, is severely impaired in metaphase oocytes following Sirt6 knockdown. Unexpectedly, we discovered that Sirt6 modulates the acetylation status of histone H4K16 as their knockdown specifically induces the hyperacetylation of H4K16 in oocytes, which may be associated with the defective phenotypes described above via altering kinetochore function. Altogether, our data reveal a novel function of Sirt6 during oocyte meiosis and indicate a pathway regulating meiotic apparatus.

In mammals, fully grown oocytes have the competence to undergo cytoplasmic and nuclear maturation, producing a haploid gamete and endowing the ability to fertilize and prepare for subsequently embryonic development^1^. In this process, the two-fold reduction of genetic materials is accomplished by a special cell division program termed meiosis. The reductional division, that occurs in meiosis I (MI), generates two pairs of sister chromatids, one of which ends up in the first polar body. The second meiotic division (MII) resembles the mitosis of somatic cells, sister chromatids segregating from each other^2^,^3^. The spindle is necessary to equally divide the chromosomes in a parental cell into two daughter cells. Deficient structure of spindle and unfaithful segregation of chromosome in meiosis could result in aneuploidy. Fertilization of these aneuploid eggs is a leading cause of spontaneous abortions or subsequently embryonic development defects if survive to term^4^,^5^. Though numerous efforts have been focus on the mechanisms involved in spindle/chromosome organization in oocytes, the underlying molecules controlling the meiotic apparatus remain to be explored.

Yeast Sir2, as a heterochromatin factor, functions in genes silence and longevity^6^. In mammals, seven members of Sir2 homolog (Sirtuin 1–7) have been identified, varying in tissue specificity, subcellular localization, enzymatic activity and targets^7^. Substantial studies have shown that Sirtuins are involved in regulating cellular metabolism, aging, and apoptosis^7^. For example, Sirt1 physically interacts with p53 and attenuates p53-mediated DNA damage-induced apoptotic response^8^. Sirt3 modulates mitochondrial intermediary metabolism and fatty-acid use in liver during fasting through deacetylating long-chain acyl coenzyme A dehydrogenase (LCAD)^9^. Compared with Sirt1 and Sirt3, not much is known about the physiology of the other Sirtuins^7^. Sirt6, the distant mammalian Sir2 homolog, was initially characterized as a nuclear ADP-ribosyltransferase^10^. Furthermore, Mostoslavsky *et al.* found that Sirt6 was a chromatin-associated protein, promoting resistance to DNA damage and suppressing genomic instability in mouse cells^11^. Recently, Sirt6 has been demonstrated to be a NAD^+^-dependent histone deacetylase. Through deacetylating histone H3 lysine 9 (H3K9), Sirt6 participates in the regulation of telomeric chromatin and cellular senescence in human U2O cells^12^. Histone H3 lysine 56 (H3K56) was also indicated as an enzymatic substrate of Sirt6 in other cell types^13^,^14^. Sirt6-mediated deacetylation of H3K56 decreases chromatin accessibility for transcription factors such as NF-kB, Foxo3, and HIF1α to their respective target promoters, thereby inhibiting the expression of their target genes^15^. In addition, under stress conditions, Sirt6 is involved in maintenance of P granules and survival of *C. elegans* by its deacetylase activity^16^. However, the potential roles for Sirt6 in meiotic oocyte have not been addressed yet. In this study, by employing morpholino knockdown, we found that Sirt6 depletion adversely impacts the maturational progression, spindle organization, and chromosome alignment in mouse oocytes.

## Results

### Cellular localization of Sirt6 during oocyte meiosis

**Figure 1:**
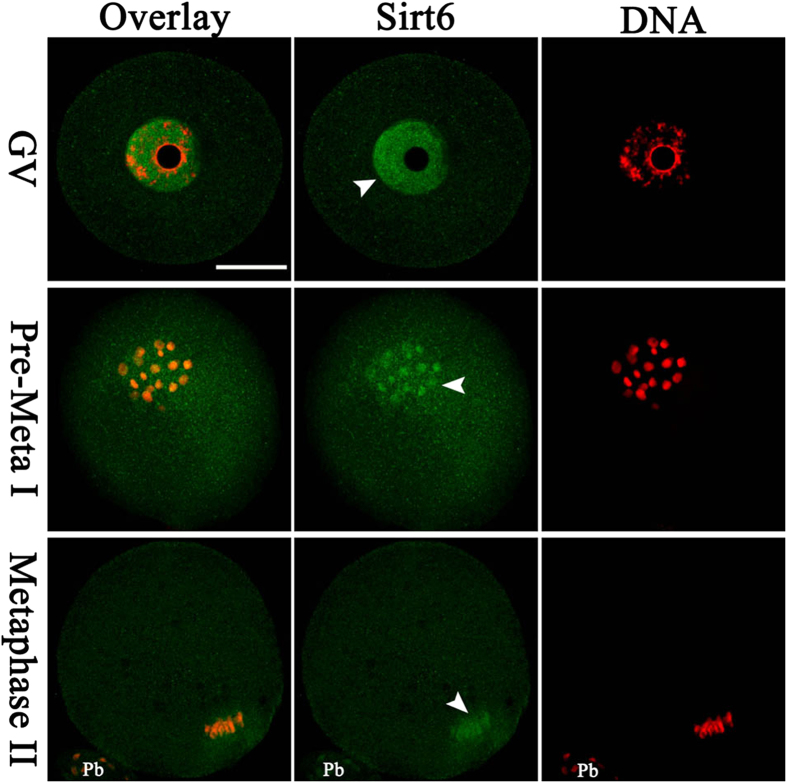
Cellular localization of Sirt6 during meiosis. Mouse oocytes at GV, pre-metaphase I, and metaphase II stages were immunolabeled with Sirt6 antibody (green) and counterstained with PI for nuclear staining (red). Arrowheads indicate Sirt6 signal. Scale bar, 25 μm.

We first examined Sirt6 localization during mouse oocyte maturation by immunofluorescent staining coupled with confocal microscopy. As show in Fig. 1, Sirt6 predominantly accumulates in the nucleus at GV stage. Accompany with the meiotic resumption, Sirt6 resides in the entire oocytes, and many of them appear to be colocalized with the chromosomes at the pre-metaphase to metaphase stages (arrowheads). The results indicate that Sirt6 may be a chromatin-associated protein in mouse oocytes, which is consistent with the data in somatic cells^11^. Such a distribution pattern prompted us to explore the potential roles of Sirt6 during oocyte maturation.

### Sirt6 depletion affects maturational progression in mouse oocytes

**Figure 2:**
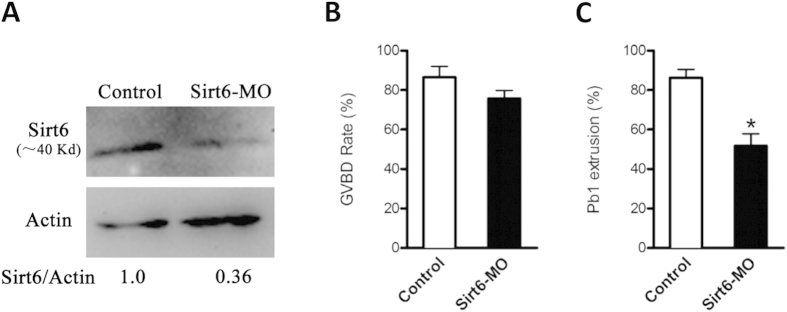
Effects of Sirt6 knockdown on oocyte maturation. Fully grown oocytes microinjected with Sirt6-MO were cultured in medium with milrinone for 20 hours to repress mRNA translation and then matured *in vitro*. A sham MO was injected as control. (**A**) The efficiency of Sirt6-MO was verified by Western blot. Band intensity was calculated using Image J software, and the ratio of Sirt6/actin expression was normalized. (**B**,**C**) Quantitative analysis of GVBD and Pb1 extrusion in control (n = 130) and Sirt6-MO (n = 125) oocytes. Bars represent means ± SD of results obtained in 3 independent experiments. *p <0 .05 vs. controls.

To investigate the function of Sirt6 in oocyte meiosis, we injected the *Sirt6*-targeting morpholino (Sirt6-MO) into fully grown oocytes. After injections, oocytes were arrested at the GV stage in medium with milrinone for 20 hours in order to block endogenous Sirt6 mRNA translation. A significant reduction of Sirt6 protein level in oocytes was confirmed by western blot (Fig. 2A). During maturation, oocytes experience germinal vesicle breakdown (GVBD), spindle assembly and chromosomes alignment at the metaphase plate (MI), first polar body (Pb1) extrusion, and then arrest at metaphase II (MII) waiting for fertilization. Our results showed that Sirt6 knockdown had no effect on meiotic resumption, as evidenced by the similar GVBD rate after 3 hours culture (75.6 ± 4.2% vs. 86.5 ± 5.5% control, p > 0.05; Fig. 2B). In contrast, the ratio of Pb1 extrusion was significantly decreased in Sirt6-MO oocytes after 14 hours *in vitro*-maturation (51.7 ± 6.2% vs. 86.2 ± 4.3% control, p < 0.05; Fig. 2C), implying the involvement of Sirt6 in oocyte maturation.

### Sirt6 is essential for spindle organization and chromosome alignment in oocytes

**Figure 3:**
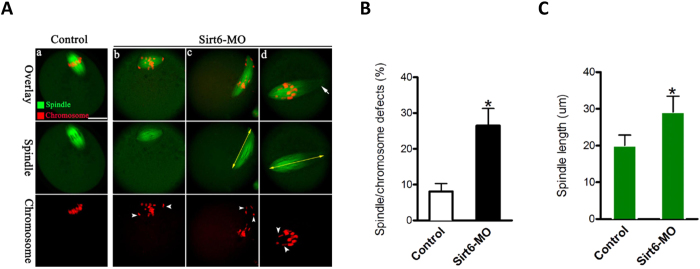
Effects of Sirt6 knockdown on spindle assembly and chromosome alignment in oocyte meiosis. (**A**) Control and Sirt6-MO oocytes were stained with α-tubulin antibody to visualize spindle (green) and counterstained with PI to visualize chromosomes (red). Control oocytes present a bipolar barrel-shaped spindle and well-aligned chromosomes on the metaphase equator (a), whereas spindle defects (arrows) and chromosomes misalignment (arrowheads) were frequently observed in Sirt6-MO oocytes (b–d). Representative confocal sections are shown. Scale bar, 25 μm. (**B**) Quantification of control and Sirt6-MO oocytes with abnormal spindle and chromosomes. (**C**) Quantitative analysis of spindle length in control and Sirt6-MO oocytes. Data are expressed as mean ± SD from 3 independent experiments in which at least 100 oocytes were analyzed. *p < 0.05 vs. controls.

Given the positioning of Sirt6 to chromosomes and its effects on maturational progression, we asked whether Sirt6 functions in the assembly of meiotic apparatus in oocytes. To gain insight into this issue, mouse oocytes from control and Sirt6-MO groups were immunolabeled with anti-tubulin antibody for spindle and counterstained with propidium iodide (PI) for chromosomes. We found a high percentage of spindle defects and chromosome congression failure in Sirt6-MO oocytes, (26.5 ± 4.8% vs. 8.1 ± 2.2% control, p < 0.05; Fig. 3A,B), displaying diverse malformed spindles (Fig. 3A, c,d, arrows) with one or several scattered chromosomes (Fig. 3A, c-d, arrowheads). In particularly, another noticeable phenotype of Sirt6-MO oocytes was spindle elongation, with average 40% longer than controls (Fig. 3A,C, arrows). By striking contrast, control oocytes at metaphase stage usually show a typical barrel-shape spindle and well-aligned chromosomes at the equator (Fig. 3A, a).

### Increased incidence of aneuploidy in Sirt6-MO oocytes

**Figure 4:**
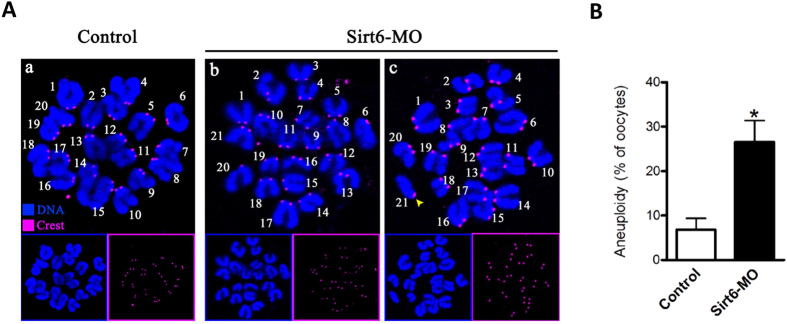
Increased incidence of aneuploidy in oocytes depleted of Sirt6. (**A**) Chromosome spread of control and Sirt6-MO MII oocytes. Chromosomes were stained with Hoechst 33342 (blue) and kinetochores were labeled with CREST (purple). Representative confocal images indicate (a) control oocytes with a normal haploid complement of 20 chromosomes, (b–c) Sirt6-MO oocytes with 21 chromosomes and premature separation of sister chromatids (yellow arrowhead). (**B**) Histogram showing the incidence of aneuploidy in control and Sirt6-MO oocytes. 50 control oocytes and 52 Sirt6-MO oocytes were analyzed respectively. Error bars indicate ± SD. *p < 0.05 vs. controls.

We postulated that spindle/chromosome anomalies in Sirt6-MO oocytes would lead to the generation of aneuploid eggs. To address this possibility, we performed the karyotypic analysis of MII oocytes by chromosome spreading combined with kinetochore labeling. As shown in Fig. 4, Sirt6 knockdown resulted in about 3-fold increase in incidence of aneuploidy eggs compared to controls (Fig. 4A shows representative images of euploidy and hyperploidy). In addition to the numerical abnormalities, we readily observed the premature separation of sister chromatids in Sirt6-MO oocytes, where the two chromatids of the chromosomes have prematurely separated before rather than during anaphase II (Fig. 4A, c, yellow arrowhead). Taking together, these observations suggest that Sirt6 knockdown disrupts the spindle assembly and chromosome movement in oocytes, consequently elevating the incidence of aneuploid eggs.

### Erroneous kinetochore-microtubule attachments in Sirt6-MO oocytes

**Figure 5:**
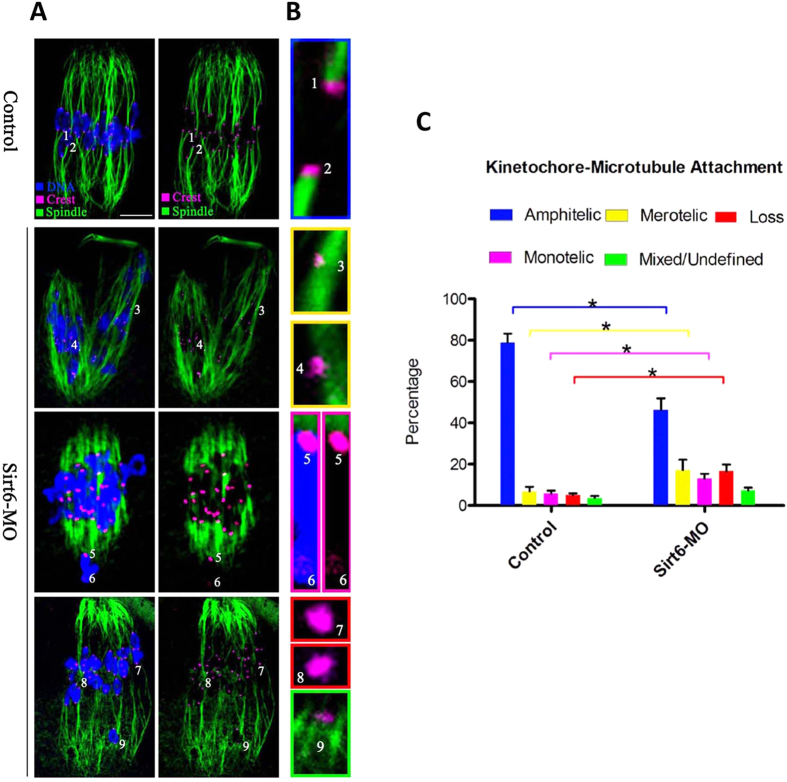
Sirt6 knockdown disrupts kinetochore-microtubule attachments in meiotic oocytes. (**A**) Control and Sirt6-MO oocytes were stained with anti-Tubulin antibody for microtubules (green), CREST for kinetochores (purple), and Hoechst 33342 for chromosomes (blue). Representative images are shown. (**B**) Magnified views for the kinetochore-microtubule attachments in the oocytes shown in (**A**). Chromosome 1 and 2 represent amphitelic attachment, chromosome 3 and 4 represent merotelic attachment, chromosome 5 and 6 represent monotelic attachment, chromosome 7 and 8 represent loss attachment, and chromosome 9 represents mixed/undefined attachment. (**C**) Quantitative analysis of K-MT attachments in oocytes as indicated. Kinetochores in regions where fibers were not easily visualized were not included in the analysis. 12 control oocytes and 10 Sirt6-MO oocytes were analyzed respectively. Scale bars, 10 μm. *p < 0.05 vs. controls.

The faithful chromosome segregation requires correct interaction between microtubules and kinetochores^17^. Harnessing the energy provided by microtubules and converting it into directional and processive chromosome movement, kinetochore plays a central role in chromosome congression and separation^18^. The high percentage of spindle/chromosome abnormalities in Sirt6-MO oocytes indicated the compromised kinetochore-microtubule (K-MT) attachments. To directly visualize the K-MT interaction, we immunostained kinetochores with CREST and microtubules with anti-tubulin antibody as described previously^19^. By confocal scanning we observed five types of K-MT attachments, including amphitelic attachment (Fig. 5B, chromosomes 1 and 2), merotelic attachment (Fig. 5B, chromosomes 3 and 4), monotelic attachment (Fig. 5B, chromosomes 5 and 6), attachment loss (Fig. 5B, chromosomes 7 and 8), as well as mix/undefined attachment (Fig. 5B, chromosomes 9). The sister kinetochores attach to microtubules emanating from opposite spindle poles, which is termed amphitelic attachment. A single kinetochore attaches to microtubules from opposite poles, giving rise to merotelic attachments^18^. When only one kinetochore attaches to one pole, the other unattached, they are called monotelic attachments^20^. Amphitelic attachment is the predominant pattern in normal cell that generate equal tension across the two kinetochores and lead to chromosomes align at the metaphase plate. While merotelic and monotelic attachments, as well as attachment loss, are the major contributors for chromosome missegregation and aneuploid^20^,^21^,^22^. By performing quantitative analysis (Fig. 5C) we found that the proportion of loss/merotelic/monotelic attachment in Sirt6-MO oocytes was increased in comparison to control oocytes (merotelic: 17.1 ± 5.0 vs. 6.8 ± 2.2% control, p < 0.05; monotelic: 13.0 ± 2.4 vs. 5.7 ± 1.5% control, p < 0.05; loss: 16.5 ± 3.3 vs. 5.0 ± 0.9% control, p < 0.05; undefined: 7.2 ± 1.4 vs. 3.6 ± 1.0% control, p < 0.05), whereas the percentage of amphitelic attachment was accordingly reduced after Sirt6 knockdown (46.2 ± 5.7 vs. 78.9 ± 4.3% control, p < 0.05). These K-MT attachment errors would inevitably result in the establishment of unstable chromosome biorientation, which is probably associated with the meiotic defects observed in Sirt6-ablated oocytes.

### Sirt6 depletion results in the hyperacetylation of histone H4K16 in oocytes

**Figure 6:**
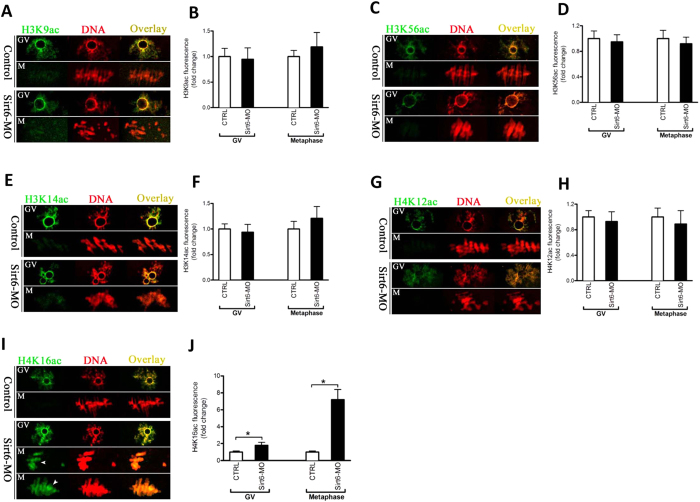
Effects of Sirt6 knockdown on the lysine acetylation of histones in mouse oocytes. Control and Sirt6-MO oocytes at GV and metaphase II stages were immunostained with an array of antibodies specifically against different acetylated histones (green), and co-stained with PI for chromosomes (red). Representative confocal images of (**A**) acetylated H3K9 (H3K9ac), (**C**) acetylated H3K56 (H3K56ac), (**E**) acetylated H3K14 (H3K14ac), (**G**) acetylated H4K12 (H4K12ac), and (**I**) acetylated H4K16 (H4K16ac) in control and Sirt6-MO oocytes. (**B**,**D**,**F**,**H**,**J**) Quantification of the data shown in panel (**A**,**C**,**E**,**G**,**I)**, respectively. At least 35 oocytes for each group were analyzed, and the experiments were repeated 3 times. Error bars indicate ± SD. *p < 0.05 vs. controls.

The effect of Sirt6 knockdown on meiotic structures prompted us to search for the underlying mechanisms that would explain these phenotypes. Sirt6 was shown to possess NAD^+^-dependent histone deacetylase activity for H3K9 and H3K56. Of note, Sirt6 functions in telomere replication via deacetylating H3K9 or K56, ultimately modulating genomic stability, chromosomal end-to-end fusions, and premature cellular senescence^12^,^13^,^14^. On the other hand, the dramatic changes in histone acetylation have been observed during mammalian oocyte maturation^23^,^24^. On the basis of these findings, we hypothesized that Sirt6 depletion might alter the status of histone acetylation in oocyte meiosis. To test this hypothesis, we first examined the global acetylation levels of H3K9 and H3K56 in Sirt6-MO oocytes. Unexpectedly, immunostaining with antibodies against specific histone residues showed that H3K9 and H3K56 acetylation remained unchanged in Sirt6-MO oocytes at both GV and metaphase stages when compared with their controls (Fig. 6A–D). Next, we further evaluate the effects of Sirt6 on the acetylation status of several other lysines on histone H3 and H4 (H3K14, H4K12, and H4K16) in oocytes. Remarkably, a specific and drastic increase in H4K16 acetylation was detected in Sirt6-MO oocytes, particularly at metaphase stage (Fig. 6I,J). By contrast, oocytes depleted of Sirt6 had little effects on both H3K14 and H4K12 acetylation (Fig. 6E–H). It is worth noting that hypoacetylation of H4K16 is essential for the establishment of functional kinetochore in both mitotic cells and meiotic oocytes^25^,^26^,^27^. Collectively, Sirt6 knockdown induced hyperacetylation of H4K16 in mouse oocytes, which may in turn perturb the chromatin conformation and kinetochore function, contributing to, at least in part, the spindle defects and chromosome misalignment during meiosis.

## Discussion

Sirt6 is a chromatin-associated protein that possesses the activity of ADP-ribosylase and NAD^+^-dependent deacetylase^28^, modulating chromatin accessibility and gene expression^15^. In the present study, we showed that Sirt6 was predominantly distributed in nucleus of immature oocytes, and then accumulated on chromosomes accompanying with meiotic resumption (Fig. 1), which is consistent with the previous findings in somatic cells^11^. Furthermore, Sirt6 knockdown in oocyte disrupted spindle formation and chromosome movement, consequently inducing the high frequency of aneuploidy (Figs 3 and 4). Faithful chromosome segregation is ensured by the bi-oriented interaction of chromosomes to the spindle through the end-on attachment of microtubules to kinetochores^29^. The kinetochore is composed of more than 100 different proteins that assembled on centromeric DNA, including inner kinetochore protein, outer kinetochore protein, and regulatory protein as well^30^. Through these multiprotein structures, kinetochore attaches chromosomes to spindle microtubules and couples power generated from microtubule polymerization and depolymerization to drive chromosomes congression^31^,^32^. Hence, those K-MT attachment errors must be corrected in normal cells because they would cause chromosome missegregation if they persisted until anaphase^29^. Here we found that Sirt6 depletion markedly disrupted the K-MT attachments during oocyte meiosis (Fig. 5). Based on these data, we propose that, in Sirt6-MO oocytes, reduction in the K-MT stability and the pulling forces across kinetochores, which could, at least in part, contribute to the chromosome alignment failure observed in our experiments. In addition, another interesting phenotype of Sirt6-depleted oocytes is the elongated spindle (Fig. 3). To date, the mechanism controlling spindle length in mammalian oocytes remains elusive^19^,^33^. Nonetheless, increasing evidence suggests that there are multiple kinetochore proteins that directly control microtubule dynamics and influence the microtubule polymerization status in mitosis^30^,^34^. How Sirt6 regulates spindle formation and its length in meiotic oocytes needs to be further investigated in the future.

In eukaryotes, the fundamental unit of chromatin is the nucleosome, which is composed of 147 base pairs of DNA and an octamer of the four core histones. A striking feature of histones, and particularly of their tails, is the large number and type of modified residues they possess, such as acetylation, phosphorylation, and methylation^35^. Among them, histone acetylation has been widely reported to participate in diverse biological events such as gene transcription, DNA replication, and chromatin condensation^35^. During maturation, oocytes display the stage-dependent and lysine residue-specific patterns of histone acetylation^23^. Moreover, inadequate histone deacetylation causes chromosome missegregation and aneuploidy in eggs, which is responsible for the subsequent embryonic development defects^36^. Specifically, hyperacetylation of H4K16 in mouse oocyte was recently demonstrated to compromise kinetochore function, generating the defective meiotic apparatus^25^,^26^,^27^. Worthy of noting, by screening the acetylation state of multiple lysine residues, we identified that Sirt6 had unique effects on the H4K16 acetylation in oocytes (Fig. 6). A plausible hypothesis is that Sirt6 knockdown induces the hyperacetylation of H4K16, which further alters K-MT connection, thereby contributing to the spindle/chromosome phenotypes we observed in oocytes. Interestingly, although Sirt6 was characterized as a NAD^+^-dependent deacetylase of histone H3K9 and H3K56 in somatic cells^12^,^13^,^14^, it seems to have little effects on their acetylation status in mouse oocytes. Instead, our results indicate that H4K16 might be a major substrate of Sirt6 in mammalian germ cells. However, owe to the limitation of oocyte number and technical reason, we have not yet been able to directly analyze the enzymatic activity of oocyte Sirt6. We also cannot rule out that Sirt6 may act on other targets in its function during oocyte maturation. Additional experiments will be required to uncover these details.

In conclusion, proper assembly of meiotic structure is crucial for maintaining oocyte quality. This study reveals that Sirt6 as a cytoskeletal regulator that is required for this process, providing a new molecular pathway controlling oocyte development and impacting women reproduction.

## Materials and Methods

All chemicals and reagents were obtained from Sigma unless otherwise stated. ICR mice were used in this study. All experiments were approved by the Animal Care and Use Committee of Nanjing Medical University and were performed in accordance with institutional guidelines.

### Antibodies

Rabbit polyclonal anti-Sirt6 (Cat#: S4197), mouse monoclonal anti-tubulin-FITC (Cat#:T6074) antibody were purchased from Sigma. Human anti-centromere CREST antibody (Cat#:15–234) was purchased from Antibodies Incorporated (Davis, CA, USA). Cy5-conjugated donkey anti-human IgG (Cat#: 709-605-149) was purchased from Jackson ImmunoResearch Laboratory (West Grove, PA, USA). FITC-conjugated goat anti-rabbit IgG was purchased from Thermo Fisher Scientific (Rockford, IL, USA). Rabbit monoclonal H4K16ac (Cat#: ab109463), rabbit monoclonal H3K56ac (Cat#: ab76307) antibodies were purchased from Abcam (Cambridge, MA, USA). Rabbit polyclonal H3K9ac (Cat#: 9671) and H4K12ac (Cat#: 06–1352) antibodies were purchased from EMD Millipore (MA, USA). And rabbit polyclonal H3K14ac (Cat#: A-4023-050) antibody was purchased from Epigentek Group Inc (Brooklyn, NY, USA).

### Oocyte collection and culture

Oocytes were retrieved from 6–8 week-old female ICR mice. 48 hours after pregnant mare serum gonadotropin (PMSG) injection, cumulus-oocyte complexes were collected by manual rupturing of antral ovarian follicles. To obtain fully grown GV oocytes, cumulus cells were removed by repeatedly pipetting. For *in vitro* maturation, GV oocytes were cultured in M2 medium under mineral oil at 37 °C in a 5% CO_2_ incubator.

### Sirt6 knockdown experiment

Sirt6-targeting morpholino (5′-CCCTGCTGCATAATTCACCGACATC-3′) was purchased from Gene Tools LLC (Philomath, OR, USA), and then diluted to 1uM as working concentration. For knockdown experiment, about 2.5 pl morpholino were injected into the cytoplasm of fully grown GV oocytes using a Narishige (Tokyo, Japan) microinjector. A non-targeting MO was injected as a control. In order to facilitate the morpholino-mediated knockdown of Sirt6 mRNA translation, oocytes were arrested at GV stage in M2 medium containing 2.5 μM milrinone for 20 hours, and then cultured in milrinone-free M2 medium for further experiments.

### Western blotting

About 100 oocytes were lysed in Laemmli sample buffer containing protease inhibitor and heated for 5 min at 100 °C. The proteins were separated by 10% SDS-PAGE and electrically transferred to PVDF membrane by constant current 200 mA. Membranes were blocked in 5% low-fat dry milk diluted by PBST for 1 h and then incubated with primary antibodies: rabbit anti-Sirt6 antibody (1:250). After washing three times in PBST, the membranes were incubated with horseradish peroxidase-conjugated secondary antibodies for 1 h. Following three times washes, the membrane was detected by an ECL Plus Western Blotting Detection System (GE Healthcare, Piscataway, NJ, USA) to visualize the protein bands. The membrane was then washed in stripping buffer and reblotted with anti-actin (1:5,000) for loading control.

### Immunofluorescence and confocal microscopy

Oocytes were fixed with 4% paraformaldehyde for 30 min and then treated in 0.5% Triton X-100 for 20 min. After being blocked in 1% BSA in PBS for 1 h, samples were incubated overnight at 4 °C with primary antibodies: anti-Sirt6 antibody, anti-H3K9ac antibody, anti-H3K14ac antibody, anti-H3K56ac antibody, anti-H4K12ac antibody, anti-H4K16 antibody, or anti-tubulin antibody. To detect kinetochores, oocytes were immunolabeled with CREST as previous described^37^. After three washes for 5 minutes each, the oocytes were labeled with secondary FITC-or Cy5-conjugated antibody for 1 hour at room temperature. Nuclear status was stained with propidium iodide (red) or Hoechst 33342 (blue) for 10 min. After briefly washed in PBS, oocytes were mounted on glass slides in a drop of antifade medium (Vectashield, Burlingame, CA, USA), and then examined with a laser scanning confocal microscope (LSM 700; Zeiss, Oberkochen, Germany). Image J software (U.S. National Institutes of Health, Bethesda, MD, USA) was used to quantify the intensity of fluorescence, as stated previously^38^.

### Chromosome spread

Chromosome spreading was performed as previously described^39^. In brief, oocytes were exposed to Tyrode’s buffer (pH 2.5) for about 30 s at 37 °C to remove zona pellucidae. After recovery in M2 medium for 10mins, oocytes were fixed in a drop of 1% paraformaldehyde with 0.15% Triton X-100 on a glass slide. After air drying, oocytes were incubated with CREST (1:500) overnight at 4 °C and then Cy5-conjugated secondary antibody for 1 h for kinetochore labeling. Chromosomes were stained with Hoechst 33342, and samples were examined under a laser scanning confocal microscope.

### Statistical analysis

Data are presented as means ± SD, unless otherwise stated. Statistical comparisons were made with Student’s test and ANOVA when appropriate. P < 0.05 was considered to be significant.

## Additional Information

**How to cite this article**: Han, L. *et al.* Sirt6 depletion causes spindle defects and chromosome misalignment during meiosis of mouse oocyte. *Sci. Rep.* **5**, 15366; doi: 10.1038/srep15366 (2015).

## References

[b1] Eppig J. J. Coordination of nuclear and cytoplasmic oocyte maturation in eutherian mammals. Reprod Fertil Dev 8, 485–489 (1996). 8870074 10.1071/rd9960485

[b2] Herbert M., Kalleas D., Cooney D., Lamb M. & Lister L. Meiosis and maternal aging: insights from aneuploid oocytes and trisomy births. Cold Spring Harb Perspect Biol 7, a017970, 10.1101/cshperspect.a017970 (2015). 25833844 PMC4382745

[b3] Jones K. T. & Lane S. I. Molecular causes of aneuploidy in mammalian eggs. Development 140, 3719–3730, 10.1242/dev.090589 (2013). 23981655

[b4] Hassold T. & Hunt P. To err (meiotically) is human: the genesis of human aneuploidy. Nat Rev Genet 2, 280–291, 10.1038/35066065 (2001). 11283700

[b5] Pont S. J. *et al.* Congenital malformations among liveborn infants with trisomies 18 and 13. Am J Med Genet A 140, 1749–1756, 10.1002/ajmg.a.31382 (2006). 16835915

[b6] Imai S., Armstrong C. M., Kaeberlein M. & Guarente L. Transcriptional silencing and longevity protein Sir2 is an NAD-dependent histone deacetylase. Nature 403, 795–800, 10.1038/35001622 (2000). 10693811

[b7] Houtkooper R. H., Pirinen E. & Auwerx J. Sirtuins as regulators of metabolism and healthspan. Nat Rev Mol Cell Biol 13, 225–238, 10.1038/nrm3293 (2012). 22395773 PMC4872805

[b8] Luo J. *et al.* Negative control of p53 by Sir2alpha promotes cell survival under stress. Cell 107, 137–148 (2001). 11672522 10.1016/s0092-8674(01)00524-4

[b9] Hirschey M. D. *et al.* SIRT3 regulates mitochondrial fatty-acid oxidation by reversible enzyme deacetylation. Nature 464, 121–125, 10.1038/nature08778 (2010). 20203611 PMC2841477

[b10] Liszt G., Ford E., Kurtev M. & Guarente L. Mouse Sir2 homolog SIRT6 is a nuclear ADP-ribosyltransferase. J Biol Chem 280, 21313–21320, 10.1074/jbc.M413296200 (2005). 15795229

[b11] Mostoslavsky R. *et al.* Genomic instability and aging-like phenotype in the absence of mammalian SIRT6. Cell 124, 315–329, 10.1016/j.cell.2005.11.044 (2006). 16439206

[b12] Michishita E. *et al.* SIRT6 is a histone H3 lysine 9 deacetylase that modulates telomeric chromatin. Nature 452, 492–496, 10.1038/nature06736 (2008). 18337721 PMC2646112

[b13] Michishita E. *et al.* Cell cycle-dependent deacetylation of telomeric histone H3 lysine K56 by human SIRT6. Cell Cycle 8, 2664–2666 (2009). 19625767 10.4161/cc.8.16.9367 PMC4474138

[b14] Yang B., Zwaans B. M., Eckersdorff M. & Lombard D. B. The sirtuin SIRT6 deacetylates H3 K56Ac *in vivo* to promote genomic stability. Cell Cycle 8, 2662–2663 (2009). 19597350 10.4161/cc.8.16.9329 PMC2728171

[b15] Kugel S. & Mostoslavsky R. Chromatin and beyond: the multitasking roles for SIRT6. Trends Biochem Sci 39, 72–81, 10.1016/j.tibs.2013.12.002 (2014). 24438746 PMC3912268

[b16] Jedrusik-Bode M. *et al.* The sirtuin SIRT6 regulates stress granule formation in C. elegans and mammals. J Cell Sci 126, 5166–5177, 10.1242/jcs.130708 (2013). 24013546

[b17] Cao Q. *et al.* A novel signal transduction pathway that modulates rhl quorum sensing and bacterial virulence in Pseudomonas aeruginosa. PLoS Pathog 10, e1004340, 10.1371/journal.ppat.1004340 (2014). 25166864 PMC4148453

[b18] Duro E. & Marston A. L. From equator to pole: splitting chromosomes in mitosis and meiosis. Genes Dev 29, 109–122, 10.1101/gad.255554.114 (2015). 25593304 PMC4298131

[b19] Ma R. *et al.* Rab5a is required for spindle length control and kinetochore-microtubule attachment during meiosis in oocytes. FASEB J 28, 4026–4035, 10.1096/fj.14-250886 (2014). 24876181 PMC5395731

[b20] Yuan J. *et al.* MAPK-activated protein kinase 2 is required for mouse meiotic spindle assembly and kinetochore-microtubule attachment. PLoS One 5, e11247, 10.1371/journal.pone.0011247 (2010). 20596525 PMC2893158

[b21] Gregan J., Polakova S., Zhang L., Tolic-Norrelykke I. M. & Cimini D. Merotelic kinetochore attachment: causes and effects. Trends Cell Biol 21, 374–381, 10.1016/j.tcb.2011.01.003 (2011). 21306900 PMC3117139

[b22] Kouznetsova A., Hernandez-Hernandez A. & Hoog C. Merotelic attachments allow alignment and stabilization of chromatids in meiosis II oocytes. Nat Commun 5, 4409, 10.1038/ncomms5409 (2014). 25007239

[b23] Gu L., Wang Q. & Sun Q. Y. Histone modifications during mammalian oocyte maturation: dynamics, regulation and functions. Cell Cycle 9, 1942–1950 (2010). 20436284 10.4161/cc.9.10.11599

[b24] Kim J. M., Liu H., Tazaki M., Nagata M. & Aoki F. Changes in histone acetylation during mouse oocyte meiosis. J Cell Biol 162, 37–46, 10.1083/jcb.200303047 (2003). 12835313 PMC2172711

[b25] Choy J. S., Acuna R., Au W. C. & Basrai M. A. A role for histone H4K16 hypoacetylation in Saccharomyces cerevisiae kinetochore function. Genetics 189, 11–21, 10.1534/genetics.111.130781 (2011). 21652526 PMC3176121

[b26] Zhang L. *et al.* Sirt2 functions in spindle organization and chromosome alignment in mouse oocyte meiosis. FASEB J 28, 1435–1445, 10.1096/fj.13-244111 (2014). 24334550 PMC3929683

[b27] Ma P. & Schultz R. M. Histone deacetylase 2 (HDAC2) regulates chromosome segregation and kinetochore function via H4K16 deacetylation during oocyte maturation in mouse. PLoS Genet 9, e1003377, 10.1371/journal.pgen.1003377 (2013). 23516383 PMC3597510

[b28] Masri S. *et al.* Partitioning circadian transcription by SIRT6 leads to segregated control of cellular metabolism. Cell 158, 659–672, 10.1016/j.cell.2014.06.050 (2014). 25083875 PMC5472354

[b29] Godek K. M., Kabeche L. & Compton D. A. Regulation of kinetochore-microtubule attachments through homeostatic control during mitosis. Nat Rev Mol Cell Biol 16, 57–64, 10.1038/nrm3916 (2015). 25466864 PMC4568440

[b30] Santaguida S. & Musacchio A. The life and miracles of kinetochores. EMBO J 28, 2511–2531, 10.1038/emboj.2009.173 (2009). 19629042 PMC2722247

[b31] Cheeseman I. M. The kinetochore. Cold Spring Harb Perspect Biol 6, a015826, 10.1101/cshperspect.a015826 (2014). 24984773 PMC4067989

[b32] Foley E. A. & Kapoor T. M. Microtubule attachment and spindle assembly checkpoint signalling at the kinetochore. Nat Rev Mol Cell Biol 14, 25–37, 10.1038/nrm3494 (2013). 23258294 PMC3762224

[b33] Howe K. & FitzHarris G. Recent insights into spindle function in mammalian oocytes and early embryos. Biol Reprod 89, 71, 10.1095/biolreprod.113.112151 (2013). 23966320

[b34] Kline-Smith S. L. & Walczak C. E. Mitotic spindle assembly and chromosome segregation: refocusing on microtubule dynamics. Mol Cell 15, 317–327, 10.1016/j.molcel.2004.07.012 (2004). 15304213

[b35] Kouzarides T. Chromatin modifications and their function. Cell 128, 693–705, 10.1016/j.cell.2007.02.005 (2007). 17320507

[b36] Akiyama T., Nagata M. & Aoki F. Inadequate histone deacetylation during oocyte meiosis causes aneuploidy and embryo death in mice. Proc Natl Acad Sci USA 103, 7339–7344, 10.1073/pnas.0510946103 (2006). 16651529 PMC1464342

[b37] Zhang D. *et al.* Intra-oocyte localization of MAD2 and its relationship with kinetochores, microtubules, and chromosomes in rat oocytes during meiosis. Biol Reprod 71, 740–748, 10.1095/biolreprod.104.028282 (2004). 15115722

[b38] Wang Q., Chi M. M. & Moley K. H. Live imaging reveals the link between decreased glucose uptake in ovarian cumulus cells and impaired oocyte quality in female diabetic mice. Endocrinology 153, 1984–1989, 10.1210/en.2011-1815 (2012). 22294751 PMC3320263

[b39] Chambon J. P., Hached K. & Wassmann K. Chromosome spreads with centromere staining in mouse oocytes. Methods Mol Biol 957, 203–212, 10.1007/978-1-62703-191-2_14 (2013). 23138954

